# Human reliability analysis in de-energization of power line using HEART in the context of Z-numbers

**DOI:** 10.1371/journal.pone.0253827

**Published:** 2021-07-01

**Authors:** Hamed Aghaei, Mostafa Mirzaei Aliabadi, Farzaneh Mollabahrami, Kamran Najafi

**Affiliations:** 1 Department of Occupational Health Engineering, School of Health, Arak University of Medical Sciences, Arak, Iran; 2 School of Public Health, Center of Excellence for Occupational Health, Occupational Health and Safety Research Center, Hamadan University of Medical Sciences, Hamadan, Iran; University of Defence in Belgrade, SERBIA

## Abstract

Investigation reveals that a high percentage of incident causes are ascribed to some forms of human error. To effectively prevent incidents from happening, Human Reliability Analysis (HRA), as a structured way to represent unintentional operator contribution to system reliability, is a critical issue. Human Error Reduction and Assessment Technique (HEART) as a famous HRA technique, provides a straightforward method to estimate probabilities of human error based on the analysis of tasks. However, it faces varying levels of uncertainty in assigning of weights to each error producing condition (EPC), denoted as assessed proportion of affect (APOA), by experts. To overcome this limitation and consider the confidence level (reliability or credibility) of the experts, the current study aimed at proposing a composite HEART methodology for human error probability (HEP) assessment, which integrates HEART and Z-numbers short for, Z-HEART. The applicability and effectiveness of the Z-HEART has been illustrated in the de-energization power line as a case study. Furthermore, a sensitivity analysis is fulfilled to investigate the validity of the proposed methodology. It can be concluded that Z-HEART is feasible for assessing human error, and despite the methodological contributions, it offers many advantages for electricity distribution companies.

## Introduction

People of each successful company are definitely its most important assets. However, detailed investigations reveal that a high percentage of incident causes have been ascribed to some forms of this element error. On the other hand, with the rapid technological innovation, the percentage of operator error in system failure tends to become conspicuous compared to technical failure. The generic term of human error as a workable label for human functions whether done or not done, is unintended by the operational system concept and negatively contributes to risk. As a result, in order to effectively prevent accidents from happening, Human Reliability Analysis (HRA) is essential as a structured way to represent unintentional operator contribution to system reliability. The main aim of HRA includes identification of human interactions in a system, hierarchical task analysis, human error quantification, and proposed reduction measures.

Due to the poor or completely absence of statistical data on human error, correct expert judgments can be collated and mapped into human error probability, through a suitable method. Human Error Reduction and Assessment Technique (HEART) as a famous HRA technique, due to its anticipative process, provide a straightforward method to estimate probabilities of human error based on the analysis of tasks. The HEART method was formed under the assumption that the joint effect of a broad selection of error producing conditions (EPCs) from the EPC reference list derives the operator’s error [1, 2]. Notwithstanding the great simplicity, comprehensibility, and user-friendliness of HEART, it faces varying levels of uncertainty in assigning of weights to each EPC, denoted as APOA, by experts. To remove the aforementioned drawback, many efforts have been made to modify the HEART method.

However, advanced approaches cannot sufficiently address different types of uncertainty. Besides, most of these approaches are still facing an open issue, namely the lack of ability to consider the confidence level (reliability or credibility) of the experts. Indeed, HEART ignores the credibility of the analyst judgment to a large extent, which may have an effect on the APOA and consequently final results.

Although in extant literature most of the recent advances in HEART are Fuzzy based, we usually use Fuzzy set to express the restriction of one thing, and the reliability of the information provided by experts also is very crucial in decision-making. We are encountering this circumstance when we are asking two exemplary experts the following question: Determine the APOA of error promoting condition “operator overload” on the subtask “apply for permit to work”. The first expert was having a PhD degree in power electrical engineering and he had been working as a consultant in electrical power transmission and distribution systems for more than 14 years. The second was having a master’s degree in safety engineering and had been working as a risk assessor and safety auditor in a regional electric company for about six years. If it is assumed that the confidence level of expert is equal and the differences in credibility are ignored, the impact of the first expert opinion on the outcome was more significant than that of the second one. However, if it is known that the second expert has a very high level of confidence in his opinion while the second expert was not, inaccurate decision information might be achieved [3]. Therefore, the APOA can be estimated much more truth by considering reliability of experts in judgment and an extension of Z-numbers can adequately accommodate the expert reliability in their preferences. It is noteworthy that in comparison to Fuzzy numbers, Z-numbers is more powerful to explain human knowledge. This supremacy is due to Z properties to explain both restriction and reliability.

Thanks to its ability to more precisely handle the expert confidence (reliability) towards their vote, the applications of Z-numbers become more and more extensive in lots of domains such as renewable energy selection [4], evolutionary games [5], psychology [6], data fusion [7], risk analysis [8, 9], control of robot [10], medicine [11, 12], and decision making process [13–16].

Up until now, according to available literature and the authors’ knowledge, the reliability or confidence of expert is not well taking into consideration in HRA techniques. Therefore, the current paper proposes a composite HEART methodology for HEP assessment which integrates HEART and Z numbers short for, Z-HEART.

The rest of the study was constructed as follows. At first, state of the art of HEART technique is reviewed. In following, basic preliminaries about classical HEART and Z-numbers are provided. Next, human error in the de-energization of the power line for maintenance was assessed to show the validity of the proposed approach. Finally, the conclusion and future directions was described.

### State of the art

After four decades of researches in the HRA reams, today these methods can be arranged into three generations [17]: in the first-generation, the methodologies mainly focus on the likelihood of human error occurrences. This generation is represented by well-established techniques, such as HEART [18], THERP [19], JHEDI [20], and SLIM [21]. The second-generation methodologies that have appeared on the basis of former category and are still being developed, put stress on cognitive information-processing to explain causes of human error. The relatively salient techniques are CREAM [22, 23], MERMOS [24], and ATHEANA [25]. The third-generation, known as next-generation methodologies, is emerging to bridge the gaps of the both previous HRA generations. The last generation employed tools such as Fuzzy logic [26, 27], Bayesian network [28], multiple criteria decision making (MCDM), Montecarlo [29], Dempster-Shafer theory [30], are applied in hybrid with already existing techniques to handle a specific challenge.

To quantify unintentional human failure probability, the dearth of empirical data on failure rate has caused challenges to current HRAs. In other words, determining the probability of the human failure is shrouded in uncertainty, because many factors may contribute to them. As a last resort in this situation, a few people with experience and knowledge specific to the process under study may be invited to provide opinions about the system being evaluated. However, uncertainty is an unavoidable part of expert elicitation process since always there is an opportunity of error, biasness, and indecisiveness in judgement.

The HEP derived in HERAT, as a widely used HRAs technique, is made up of three factors (GTT, EPC, and APOA). While, the two first factors are relatively structured affair, probability of human error to a great extent depends on the accuracy of experts’ judgment during APOA derivation. In the APOA estimation, the importance of each chosen EPC on the task under study is elicited based on expert opinion. APOA as a value, which varying from 0 to 1 (0%–100%), reflect how much EPCs influence on human error and has to be decided by the analyst. However, there are some vagueness in obtained information in two aspects of experts’ opinions and sureness of expert in opinions. Thus, because of experts’ unreliable judgment, it imposed some shortages to the analysis. By the other hand, HEART does not assist analyst in a concrete manner for appraisal of APOA. However, the matter of uncertainty in APOA appraisal has not gone unnoticed in safety and HRA scholars have been proposed several methodologies to rectify this problem in many different ways. To overcome the inconsistencies related to expert judgments, for example Casamirra et al. amalgamate Fuzzy theory, HEART, and FTA analysis to study human error probability in the irradiation facilities [31]. Castiglia and Giardina [32] applied Fuzzy set concept and conventional HEART to derive HEP in pressure CV (control valve) maintenance in hydrogen stations. Akyuz and Celik [33] suggested a hybrid HEART based on AHP to obtain the EPCs proportion of effect in calculation operator error probabilities. These authors also presented an extended methodology of HEART which integrated with type-2 Fuzzy to cope with the experts’ elicitations uncertainty in the APOA appraisal [34]. Maniram Kumar et al. [35] used Fuzzy logic combining with HEART to overcome the inaccuracy of experts’ preferences in assigning the relative strength of EPCs over GTT. Mirzaei Aliabadi [27] proposed a human error analysis composite HEART and Intuitionistic Fuzzy to find HEP in furnace start-ups tasks. Using Fuzzy HEART, Mirzaei Aliabadi et al. [36] assess the probability of maintenance error in catalyst replacement of process refinery. Based on Dempster–Shafer (D-S) theory, Islam et al. [30] introduced a developed HEART, to fuse opinions of exert panel regarding the importance of each chosen EPC on the maintenance task of a condensate pump. Wang et al. [37] firstly put forward a new HEART model to assess human error probability in high-speed railway dispatching tasks with amalgamation of original HEART and Fuzzy analytic network process (FANP). To appraisal human error in daily control procedure of the steam boiler Can et al. provided an extended HEART based on advanced version of decision making trial and evaluation laboratory (AVDEMATEL) integration. Guo and Sun [38] made an endeavor to quantify the APOA values in assessing human error in the aircraft manipulation process using HEART combined with improved analytic hierarchy process (IAHP) method.

It is highly difficult to find a research that specifically composite Z-numbers with HRAs technique to handle the confidence level of expert.

Based on the relevant literature review conducted on the abovementioned works, it was observed that this work contributes to the context of maintenance error assessment as follows. This is the first time to merge conventional HEART with Z-numbers, in order to properly obtain EPCs proportion of effect. Besides, in the prevailing literature, there has been no attempt to analysis human reliability in de-energization of the power line in electrical maintenance. Hence, the current article aims to remedy the gap in this particular scope using the proposed novel Z-numbers-based HEART approach (shortly, Z-HEART).

## Materials and methods

### Ethical statement

The study was approved by the ethics committee of Hamadan University of Medical Sciences (Ethics Committee No: IR.UMSHA.REC.1398.1087).

### HEART technique

The HEART technique was first introduced in 1985 by Williams and 3 years later was outlined in detail [39]. Basically, this technique is employed to assess human tasks with given number values for human error (Generic Error Probability) specific to tasks and for environment in which operators perform (context). Considering the selected values of tasks and error producing conditions (as context) the formula of final probabilities of human error are computed. In a nutshell, the general application consisted of the following steps:

1. Task analysis
2. Attribute correspondent generic task type
3. Assign the EPCs detriment the activity
4. Assessed proportions of affect (APOA) of the assigned EPCs
5. Compute the final HEP.

Given above, HEP can be found as [36]:

$$Final\;HEP={GEP}_{j}\times Partial\;HEP$$ (1)

$$Partial\;HEP=\prod_{i}^{38}{APOA}_{i}\times \left({EPC}_{i}-1\right)+1\;for\;i=1,2,3,\ldots ,n;n\leq 38$$ (2)

Where *EPC*_*i*_ = EPC for the *i*th condition, Table 1; *APOA*_*i*_ = APOA for *i*th condition; *HEP* = Final HEP; *GEP*_*j*_ = GEP (the Generic Error Probability is given to each GTT, Table 2) related with the to be evaluated task.

**Table 1:** Generic Task Type descriptions and GEP values.

GTT ID	Generic Task Type (GTT)	GEP	5th-95th percentile bound
A	Totally unfamiliar, performed at speed with no real idea of the likely consequences	0.55	(0,35–0,97)
B	Shift or restore system to a new or original state on a single attempt without supervision or procedures	0.26	(0,14–0,42)
C	Complex task requiring a high level of comprehension and skill	0.16	(0,12–0,28)
D	Fairly simple task performed rapidly or given scant attention	0.09	(0,06–0,13)
E	Routine, highly practiced, and rapid task involving a relatively low skill level	0.02	(0,07–0,045)
F	Restore or shift the system to an original or new state following procedures, with some checking	0.003	(0,0008–0,007)
G	Completely familiar, well-designed, highly practiced, and routine task occurring several times per hour, performed to the highest possible standards by highly motivated, highly trained and experienced persons, totally aware of the implications of failure and having the time to correct potential errors but without the benefits provided by significant job aids	0.0004	(0,00008–0,009)
H	Respond correctly to system commands even when there is an augmented or automated supervisory system providing an accurate interpretation of the system state	0.00002	(0,000006–0,009)
M	Miscellaneous task for which no description can be found	0.03	(0.008–0.11)

**Table 2:** EPC definition and related maximum value.

ID	Error Producing Condition	Weight
1	Unfamiliarity with a situation which is potentially important but which only occurs infrequently or which is novel	×17
2	A shortage of time available for error detection and correction	×11
3	A low signal-noise ratio	×10
4	A means of suppressing or over-riding information or features which is too easily accessible	×9
5	No means of conveying spatial and functional information to operators in a form which they can readily assimilate	×8
6	A mismatch between an operator’s model of the world and that imagined by a designer	×8
7	No obvious means of reversing an unintended action	×8
8	A channel capacity overload, particularly one caused by simultaneous presentation of no redundant information	×6
9	A need to unlearn a technique and apply one which requires the application of an opposing philosophy	×6
10	The need to transfer specific knowledge from task to task without loss	×5.5
11	Ambiguity in the required performance standards	×5
12	A mismatch between perceived and real risk	×4
13	Poor, ambiguous or ill-matched system feedback	×4
14	No clear direct and timely confirmation of an intended action from the portion of the system over which control is to be exerted	×4
15	Operator inexperience (e.g., a newly-qualified tradesman, but not an "expert")	×3
16	An impoverished quality of information conveyed by procedures and person/person interaction	×3
17	Little or no independent checking or testing of output	×3
18	A conflict between immediate and long-term objectives	×2.5
19	No diversity of information input for veracity checks	×2.5
20	A mismatch between the educational achievement level of an individual and the requirements of the task	×2
21	An incentive to use other more dangerous procedures	×2
22	Little opportunity to exercise mind and body outside the immediate confines of a job	×1.8
23	Unreliable instrumentation (enough that it is noticed)	×1.6
24	A need for absolute judgments which are beyond the capabilities or experience of an operator	×1.6
25	Unclear allocation of function and responsibility	×1.6
26	No obvious way to keep track of progress during an activity	×1.4
27	A danger that finite physical capabilities will be exceeded	×1.4
28	Little or no intrinsic meaning in a task	×1.4
29	High-level emotional stress	×1.3
30	Evidence of ill-health amongst operatives, especially fever	×1.2
31	Low workforce morale	×1.2
32	Inconsistency of meaning of displays and procedures	×1.2
33	A poor or hostile environment (below 75% of health or life-threatening severity)	×1.15
34	Prolonged inactivity or highly repetitious cycling of low mental workload tasks (for 1st half hour)	×1.1
34	(Thereafter)	1.05
35	Disruption of normal work-sleep cycles	×1.1
36	Task pacing caused by the intervention of others	×1.06
37	Additional team members over and above those necessary to perform task normally and satisfactorily (per additional man)	×1.03
38	Age of personnel performing perceptual tasks	×1.02

### Basic preliminaries of Z numbers

**Fig 1:**
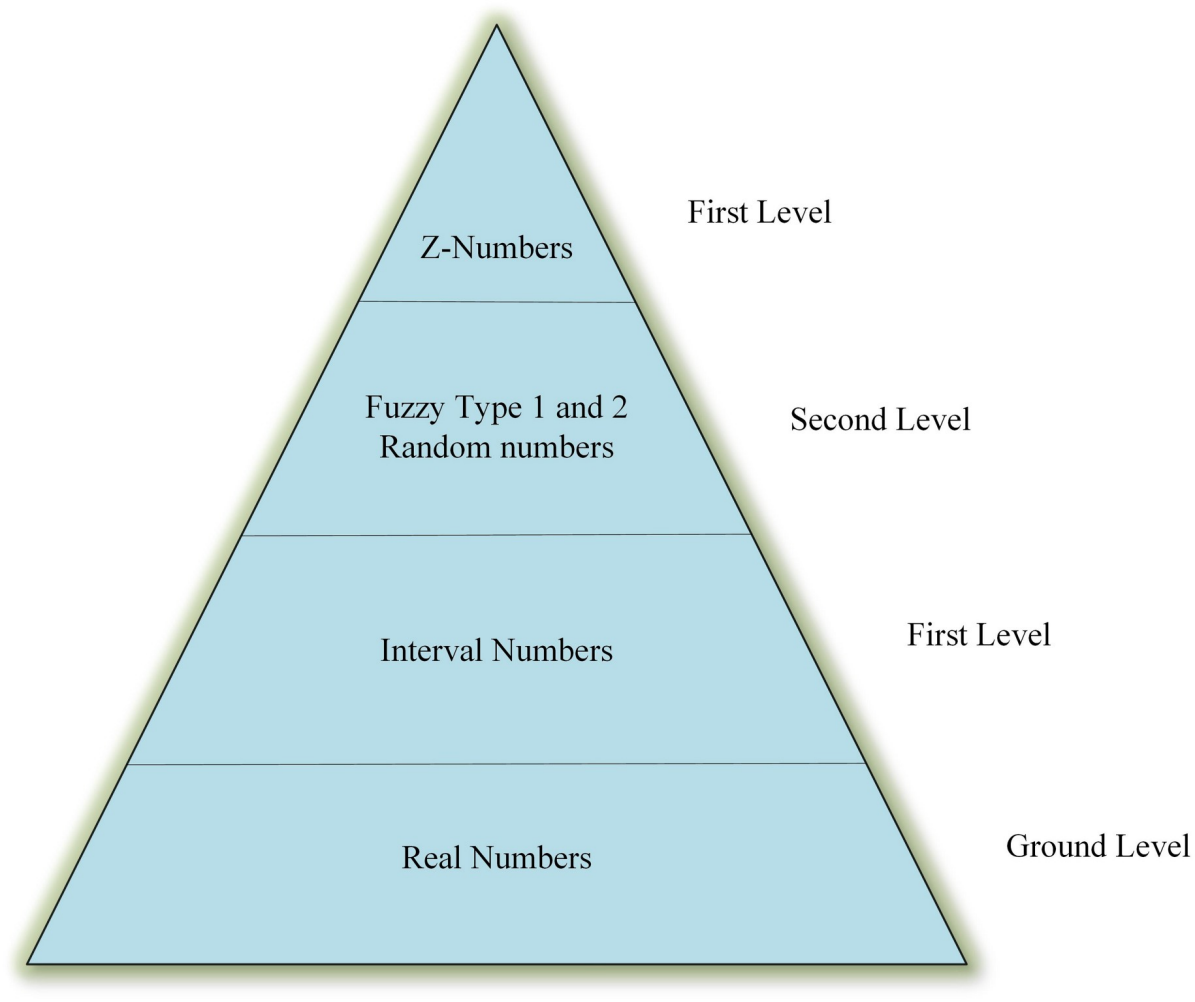
Hierarchical structure of uncertainty.

Z-numbers, as an extension of Fuzzy set but more effective, opened doors to a new field developed by Zadeh [40] to provide a computation basis for uncertain information. Information uncertainty as an inevitable element of any decision-making process has a hierarchy, often consists of the following levels of confinements that handles uncertain information: confidence interval, type-1 Fuzzy sets, type-two Fuzzy sets (IT2FS), and Z-number. Fig 1 shows the hierarchy levels. The Z-number is an ordered pairwise of Fuzzy numbers denoted as $Z=\left(\tilde{A},\tilde{R}\right),$ where $\tilde{A}$ is a Fuzzy restriction on the values that *X*_*A*_ can take, and $\tilde{R}$ is the reliability of value $\tilde{A}$ [41]. In fact, the probability of $\tilde{A}$ value is estimated by $\tilde{R}$. For uncertain variable X in particular, a further definition can be displayed as

$$Prob\left(X\;\text{is}\;\tilde{A}\right)is\;\tilde{R},$$

**Fig 2:**
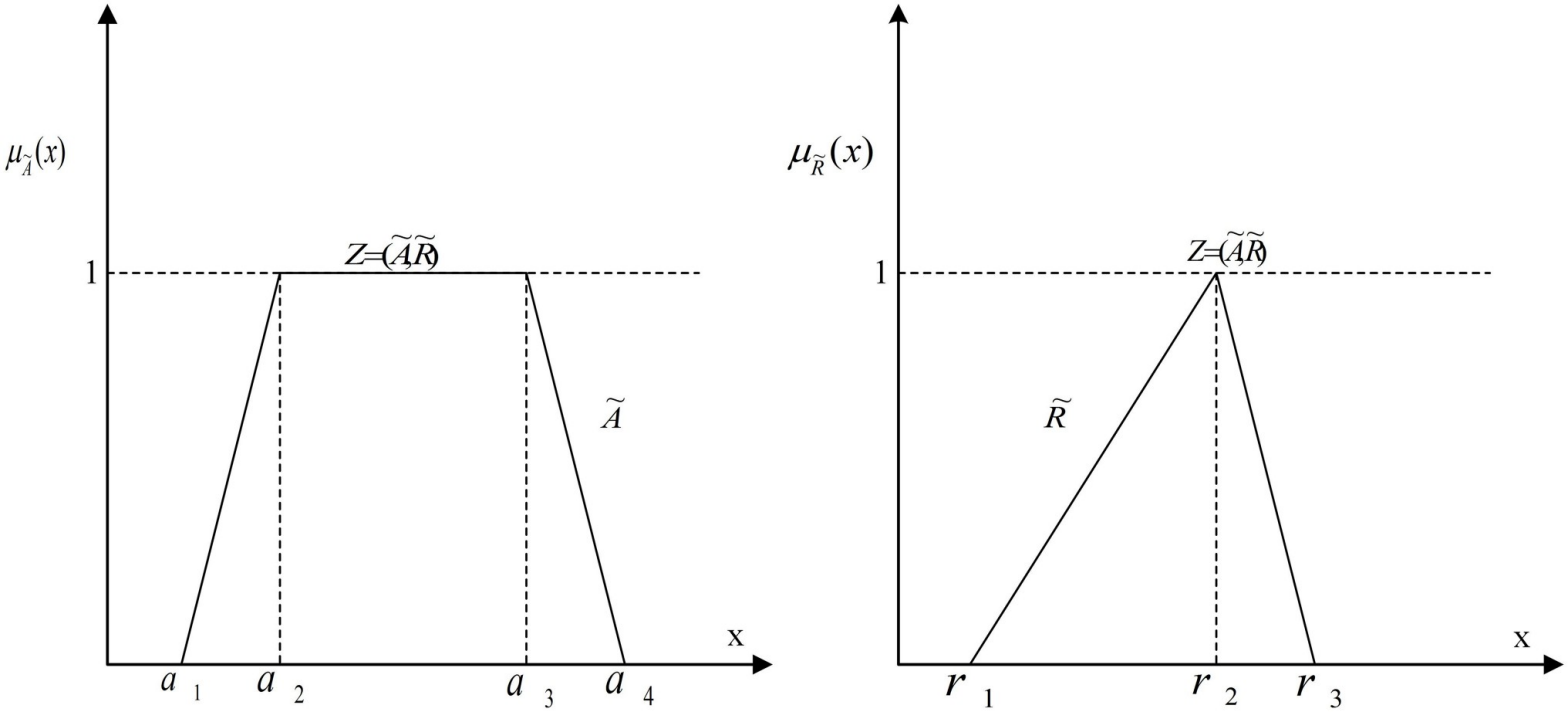
A simple Z-number, $Z=(\tilde{A},\tilde{R})$. $\tilde{A}$ is a fuzzy restriction and $\tilde{R}$ is the reliability of $\tilde{A}$ value.

In which, Prob presents the probability value. As an example, the sentence “operator inexperience effect the check in no voltage condition is likely to be high” is a Z concept. The effect of the check in no voltage condition is a Fuzzy number. The effect of operator inexperience on the check in no voltage condition may be mildly high. But, “likely” is the probability of this event. It may affect the check in no voltage condition other levels in some context. In Fig 2 a simple Z-number is illustrated. $\tilde{A}$ and $\tilde{R}$ are trapezoid and triangular Fuzzy numbers which can be defined by $\tilde{A}=\left(a_{1},a_{2},a_{3},a_{4}\right)$ and $\tilde{R}=\left(r_{1},r_{2},r_{3}\right)$, respectively [42].

Given Z-number $Z=(\tilde{A},\tilde{R})$, suppose $\left\{(\tilde{A}=\left(x,\mu_{\tilde{A}}(x))\left|x\in \left[0,1\right]\right.\right)\right\}$ and $\left\{(\tilde{R}=\left(x,\mu_{\tilde{R}}(x))\left|x\in \left[0,1\right]\right.\right)\right\}$. Parameters $\mu_{\tilde{A}}$ and $\mu_{\tilde{R}}$ indicate the trapezoidal and triangular Fuzzy membership functions, respectively. Firstly, the crisp centroid (center of gravity, COG) value of $\tilde{R}$ are calculated to transform reliability (right side of Z number) in to a crisp value α as follows [43]:

$$\alpha =\frac{\int \mu_{\left(R\right)}\left(x\right)xdx}{\int \mu_{\left(R\right)}\left(x\right)dx}$$ (3)

Then, add the weights of the right side (*α*) to the left side (expert opinion). So, the weighted reliability is illustrated as [43]:

$${\tilde{Z}}^{a}=\left\{\left(x,\mu_{{\tilde{A}}^{a}}(x)\right)\left|\mu_{{\tilde{A}}^{a}}\left(x\right)=a\mu_{\tilde{A}}(x)\right.,x\in \left[0,1\right]\right\}$$ (4)

Since the maximum height of the ${\tilde{Z}}^{a}$ is α not 1, it cannot be considered as a normal Fuzzy number. According to fuzzy expectation of a fuzzy number it can be converted to regular Fuzzy number by considering reduction coefficient $\sqrt{a}$, denoted as ${\tilde{Z}}^{\prime }$ [43]:

$${\tilde{Z}}^{\prime }=\left\{\left(x,\mu_{{\tilde{Z}}^{\prime }}(x)\right)\left|\mu_{{\tilde{Z}}^{\prime }}\left(x\right)=\mu_{\tilde{A}}\left(\frac{x}{\sqrt{a}}\right),x\in \left[0,1\right]\right.\right\}$$ (5)

**Fig 3:**
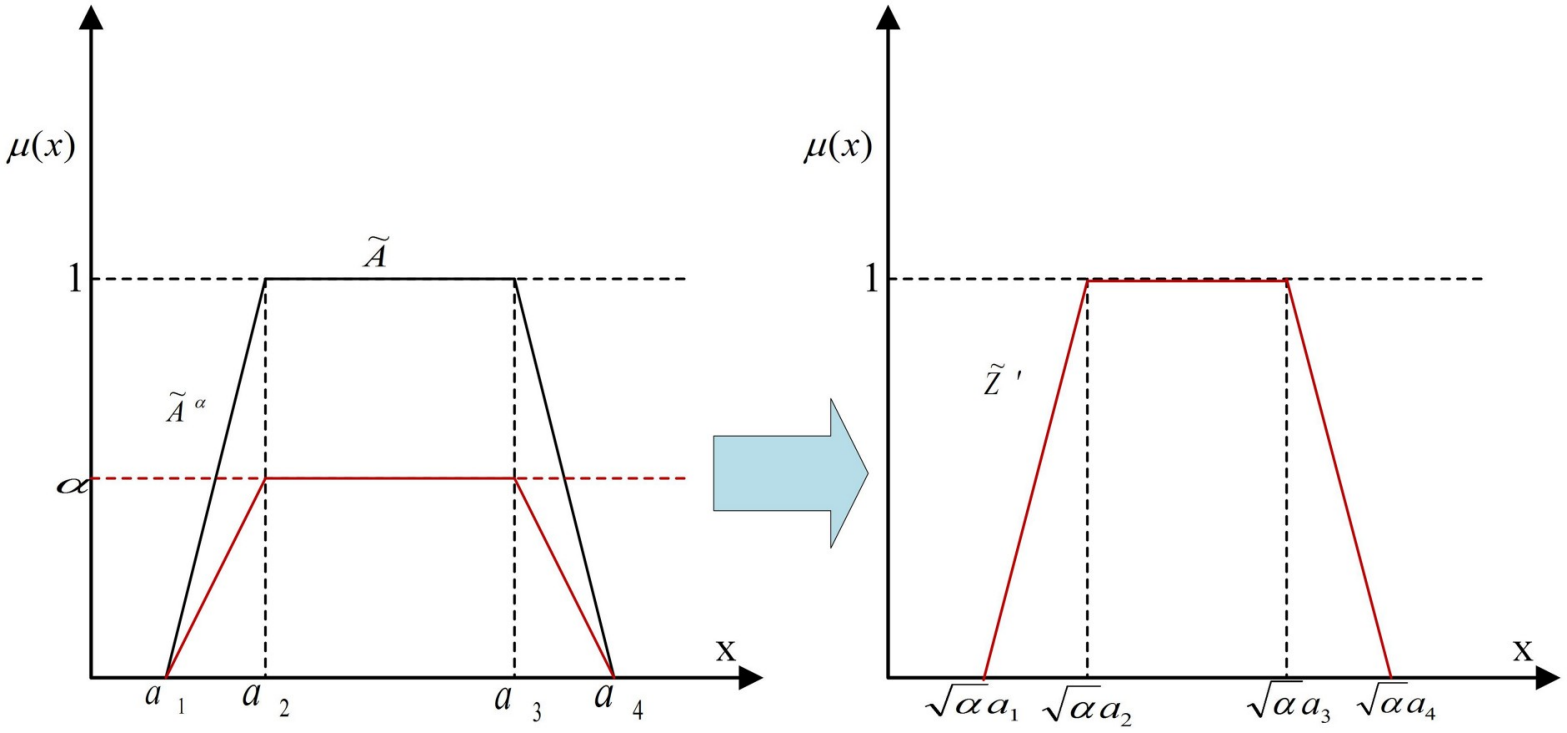
Transforming of Z-number to Fuzzy number. *α* is crisp value of reliability side of Z-number. $\tilde{A}$ is the left side of Z-number and ${\tilde{Z}}^{\prime }$ is the final normal Fuzzy number.

It has been demonstrated that the Fuzzy expectation of ${\tilde{Z}}^{\prime }$ is equivalent to ${\tilde{Z}}^{a}$. Fig 3 summarizes the transformation method. For details of the proof is referred to Kang et al. [43].

### Proposed Z-HEART

**Fig 4:**
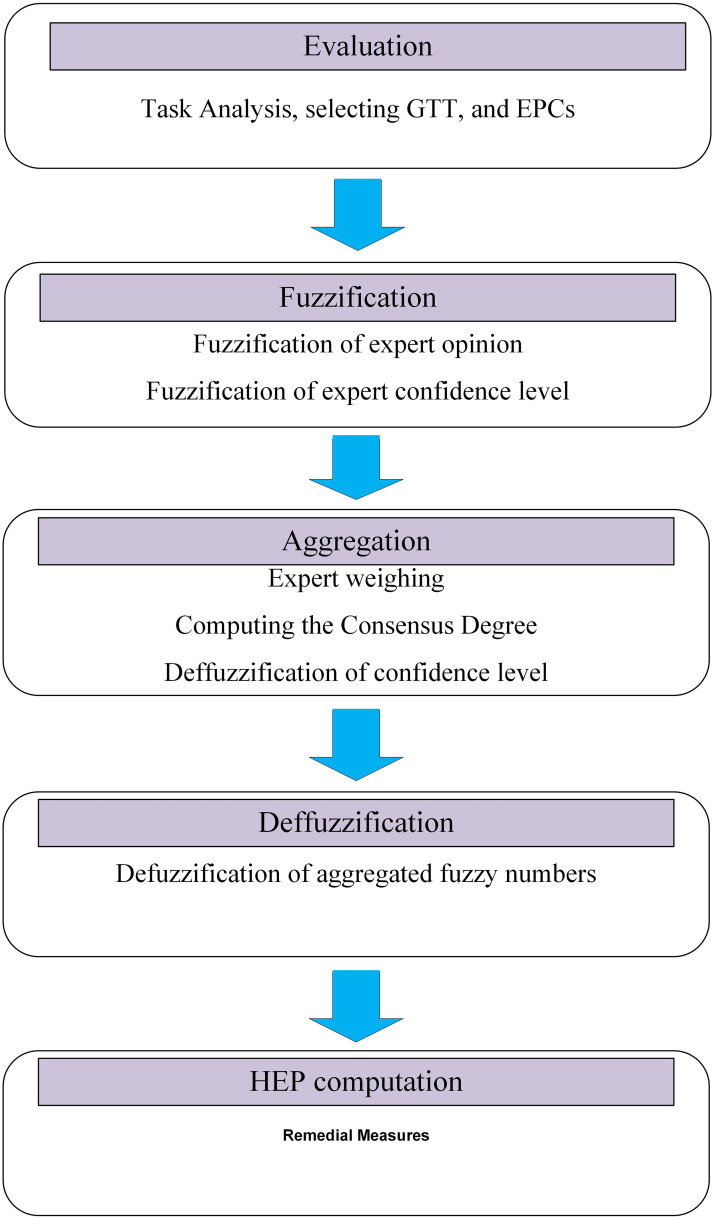
Algorithm of the proposed approach.

According to the previous section, an integrated technique based on the HEART and Z-numbers short for Z-HEART is proposed. To facilitate the application, the algorithm of this technique is summarized below. Excel software (Microsoft Excel (2007)) was used to perform all analysis. The procedure of this algorithm is depicted in Fig 4.

### Task analysis

The first and common step in HRA methods is to specify the human involvement in the sociotechnical system, which obtained by the application of hierarchical task analysis. This step, among other things, determines the human’s tasks, practices to be pursued and essential tools.

### Attribute GTT and EPCs to each identified task

Finding the best fit generic task type (from nine general tasks in Table 1) to task is being assessed and next selecting the generic error probability from proposal range values, is the main goal of this step. For instance, responding to “check in no voltage condition” must use Table 1, to select the fittest GTT. Considering the fact that it is not a complex duty, E could be related, with a baseline human error probability of 0.02 or 2 errors in 100 opportunities. Afterwards, the assessor ascertains which human error forcing context under the HEART methodology named error-producing conditions (EPC) that related to be evaluated task. As it apparent from Table 2, each of them has a specific weight. If for a given task more than one EPC can be considered, next step, APOA calculation is required.

### Assessed proportions of affect (APOA) of the assigned EPCs

In this step, the importance of each chosen EPC on the task under analysis is elicited based on expert opinion. APOA as a value, which varies from 0 to 1 (0%–100%) reflects the influence of EPCs on human error, and has to be decided by the analyst. However, there are some vagueness in obtained information in two aspects of experts’ opinions and sureness of expert in opinions. Z-number could be combined with HEART to overcome this shortcoming in APOA evaluation. The suggested APOA appraisal in the context of Z-number is summarized as below:

#### Stage 1: Domain expert investigation

The purpose of this stage is to elicit two types of information from expert using Z-number: the opinion about the occurrence probability (importance of each chosen EPC on the tasks), and subjective reliability level (confidence level or credibility) of what they are saying. This information was synthesized to reach the most reasonable judgment. In addition, the effect of difference in quality of expert is presented by expert judgment ability level (from 0–1), calculated on the basis of criteria set out in Table 3. In this study, corresponding Z-applied set was utilized for estimating of APOA, which are illustrated in Table 4.

**Table 3:** Score rating of different experts.

Factors	Classify	Score
Job position	Operator	1
	Technician	2
	Engineer	3
	Manage	4
	Director	5
Service time	≤5	1
	6 to 9	2
	10 to 19	3
	20 to 29	4
	≥30 year	5
Educational level	High school	1
	Higher national diploma	2
	BSc	3
	MSc	4
	PhD	5

**Table 4:** Corresponding Z-applied set.

	Linguistic term	Scale
Conversion scale for restriction (expert opinions)	Very Low (VL)	(0, 0, 0.1, 0.2)
	Low (L)	(0.1, 0.2, 0.2, 0.3)
	Mildly Low (ML)	(0.2, 0.3, 0.4, 0.5)
	Medium (M)	(0.4, 0.5, 0.5, 0.6)
	Mildly High (MH)	(0.5, 0.6, 0.7, 0.8)
	High (H)	(0.7, 0.8, 0.8, 0.9)
	Very High (VH)	(0.8, 0.9, 1.0, 1.0)
Conversion scale for reliability	Very Low (VL)	(0, 0.1, 0.2)
	Low (L)	(0.1, 0.25, 0.4)
	Medium (M)	(0.3, 0.5, 0.7)
	High (H)	(0.6, 0.75, 0.9)
	Very High (VH)	(0.8, 0.9, 1.0)

After that, for each EPC, transform subjective reliability level to *α*, add it to the expert opinion (left side of Z-number), and subsequently was converted the results to regular Fuzzy number. The output of this stage is to be aggregated in onward stages to get a single number.

#### Stage 2: Aggregation

Because of the different opinions on the probability, fuzzified linguistic terms gathered from the previous stage were cumulated in this stage to provide a single probability. For this reason, the algorithm can be briefly described [44].

1. Computing the similarity degree
  The degree of similarity *S*(*A*_*i*_, *A*_*j*_) between the opinions *A*_*i*_ and *A*_*j*_ of two experts was evaluated. Given *A*_*i*_ = (a_1_, a_2_, a_3_, a_4_) and *A*_*j*_ = (b_1_, b_2_, b_3_, b_4_) are the two Z standard trapezoid Fuzzy numbers, similarity scores were estimated in accordance with the following equation [45]:
$$S\left(A_{i},A_{j}\right)=1-\frac{1}{4}\sum_{i=1}^{4}\left|a_{i-}b_{i}\right|$$ (6)
2. Computing the Average Agreement AA (Ei) degree for each of the experts [45]:
AAEi=1n-1∑i≠jj=1nSij(Ai,Aj) (7)
3. The Relative Agreement RA (E_i_) degree, of all experts was found as follows [45]:
RA(Ei)=AA(Ei)∑i=1nAA(Ei) (8)
4. Likewise, the consensus coefficient degree C (E_i_) of the experts was yielded as follows [45]:
$$C\left(E_{i}\right)=\beta \;\text{W}(E_{i})+(1–\beta )\times RA(E_{i})$$ (9)
  where, *β* ∈ [1,0], as dominance factor shows the priority of judgment ability level of experts over relative agreement.
5. Computing aggregation results, according to the following equation [45]:
$$R_{AGZ}=C\left(E_{1}\right)\times {{\tilde{Z}}^{\prime }}_{1}+\text{C}\left(E_{2}\right)\times {{\tilde{Z}}^{\prime }}_{2}+\ldots +C(E_{i})\times {{\tilde{Z}}^{\prime }}_{i}$$ (10)
  where, *R*_*AGZ*_ is an aggregate Fuzzy number of EPC, and ${{\tilde{Z}}^{\prime }}_{i}$ is the regular Fuzzy number (Eq 5).

#### Stage 3: Defuzzification process

The aim of this stage was to determine a crisp value as the representative of the aggregate Fuzzy number. Defuzzification of aggregated trapezoidal Fuzzy number *A*_*i*_ = (a_1_, a_2_, a_3_, a_4_) could be described as follows:

$$X^{*}=\frac{\int_{a_{1}}^{a_{2}}\frac{x-a_{1}}{a_{2}-a_{1}}xdx+\int_{a_{2}}^{a_{3}}xdx+\int_{a_{3}}^{a_{4}}\frac{a_{4}-x}{a_{4}-a_{3}}xdx}{\int_{a_{1}}^{a_{2}}\frac{x-a_{1}}{a_{2}-a_{1}}dx+\int_{a_{2}}^{a_{3}}dx+\int_{a_{3}}^{a_{4}}\frac{a_{4}-x}{a_{4}-a_{3}}dx}=\frac{1}{3}\left(\frac{{\left(a_{4}+a_{3}\right)}^{2}-a_{4}a_{3}-{\left(a_{1}+a_{2}\right)}^{2}+a_{1}a_{2}}{a_{4}+a_{3}-a_{1}-a_{2}}\right)$$ (11)

### Computing the final HEP

After calculating APOAs based on Z-number, the Eqs 1 and 2 are applied to obtain human error probability of all sub-tasks.

### Human error probability in de-energization of power line

Performing maintenance on overhead power lines really is a daring job that requires comprehensive hazard awareness of the performed tasks and safe working practices. In the United States, during the years 2003–2007, exposure with overhead power lines resulted 43% of all on-the-job fatal electrocutions [46]. Statistical records of eight years, in Iran show that from 2007 to 2015, 119 electrical accidents happened and worker failure was found as the cause of 75 percent of them [47]. In working on electrical lines, where coming in contact with live line can lead to death or severe injuries, the most basic safety approach is to de-energize lines. Deenergizing, also known as clearing, requires more than merely turning a switch off. In order to prevent this accident, strict safety measures must be provided before beginning work. Given the hazardous nature involved, a human failure in de-energization process can lead to severe injuries and fatalities. Hence, it is expected human reliability analysis help improve safety by providing procedural recommendations. The case study was conducted in an electrical distribution company in Iran. In this company, more than 35 maintenance groups are daily involved with the maintenance and most of these groups have three maintainers.

### Task analysis

De-energization power line can be decomposed into five main tasks, on the basis of hierarchical task analysis, including:

1. Request to de-energize: The maintenance crew shall make a request of the operation unit to de-energize a given portion of distribution line.
2. De-activation: The most basic action is to open all energy control devices feeding the sections of the distribution line, which workers may be exposed, operation crew.
3. Test/checks: Conduct voltage measurements and visual inspections to verify all line is de-energized. To verify that the line is de-energized and safe, voltage measurements shall be made at the disconnected line.
4. Discharge: Discharge any hazardous energy accumulated in the de-energized lines. An extremely simple way to discharge an overhead line without leaving the ground is wire gun tester application.
5. Install temporary grounds: Safety temporary grounds shall be installed to ensure the presence of an equipotential working zone.

Distribution of main tasks made up 26 subtasks, as summarized in Table 5. The potential of human error is calculated for all of these subtasks, which provided by HTA.

**Table 5:** Hierarchical task analysis for the de energization power line.

Subtask ID	Task description
**DE1**	**Request to de-energize**
T1	Area authority make work order
T2	Apply for permit to work
T3	Identify and determine location of de-energization
**DE2**	**De-energization**
T4	Wear appropriate PPE
T5	Feeder disconnection
T6	Lock and tag
T7	Communicate the disconnection to operation unit
**DE3**	**Perform voltage test**
T8	Wear appropriate PPE
T9	Ensure that instrument is operable
T10	Check in no voltage condition
T11	Retest that instrument is operable
T12	Informed the operation unit of testing results
**DE4**	**Discharge the de-energized line**
T13	Install the grounding rod in opposite side of shooting
T14	Open the spool of fuse wire from shooting site toward grounding rod
T15	Fasten one end of the fuse wire to grounding rod and the other end to spare arrow.
T16	Insert the spare arrow in gun tester
T17	Ensure that the opened fuse wire is not put behind the shooter or other crew members
T18	Shooting and interpretation of outcomes
T19	Informed the discharging to operation unit
**DE5**	**Install temporary grounds**
T20	Sitting the location of inserting ground electrode
T21	Visually inspect any defect in safety ground wire which is to be applied.
T22	Ensure that all not necessary maintainers have been cleared from the working zone
T23	Fasten the ground end of the safety temporary ground electrode first
T24	Contact and tighten the grounding clamp from the closest to the farthest phase one at a time with the phase end of the safety temporary ground.
T25	Install temporary ground, also at the other end of de energized section
T26	Informed the installation temporary grounds to operation unit

### Attribute GTT and EPCs to each identified task

The subtask of de-energization of power line is assigned to suitable GTTs based on duty descriptions. Afterward, based on specific operational conditions, a set of associated EPCs is selected of supplied EPCs. The results are presented in Table 6.

**Table 6:** Human error probability results.

Subtask	Generic task type	Generic error probability	Error producing conditions	Human error probability	
**1**				Z based	Fuzzy set based
T1	G	0.0004	EPC 8, 16, 29	1.95E-03	3.47E-03
T2	G	0.0004	EPC 2, 13	3.19E-03	5.78E-03
T3	F	0.003	EPC 3, 18, 35	1.36E-02	2.18E-02
**2**					
T4	G	0.0004	EPC 8, 17	1.62E-03	2.16E-03
T5	F	0.003	EPC 2, 15, 17	1.88E-02	3.56E-02
T6	E	0.02	EPC 2, 29, 31	6.54E-02	8.45E-02
T7	G	0.0004	EPC 3, 16	1.77E-03	2.60E-03
**3**					
T8	G	0.0004	EPC 8, 15, 31	1.99E-03	2.91E-03
T9	E	0.02	EPC 15, 17	3.96E-02	5.56E-02
T10	E	0.02	EPC 8, 17, 23	7.53E-02	1.21E-01
T11	F	0.003	EPC 11, 33, 35	8.09E-03	1.09E-02
T12	G	0.0004	EPC 8, 23	1.38E-03	1.70E-03
**4**					
T13	G	0.0004	EPC 11, 17	1.47E-03	1.96E-03
T14	E	0.02	EPC 15, 33	3.34E-02	3.92E-02
T15	G	0.0004	EPC 13, 17	1.12E-03	1.60E-03
T16	G	0.0004	EPC 13, 23	8.44E-04	9.87E-04
T17	G	0.0004	EPC 5, 31	9.67E-04	1.14E-03
T18	F	0.003	EPC 8, 15, 16	1.41E-02	2.11E-02
T19	G	0.0004	EPC 2, 23, 33	1.63E-03	2.31E-03
**5**					
T20	H	0.00002	EPC 15, 33	3.38E-05	3.78E-05
T21	G	0.0004	EPC 2, 26, 33	1.48E-03	2.03E-03
T22	F	0.003	EPC 2, 15	1.18E-02	1.73E-02
T23	G	0.0004	EPC 6, 15	1.09E-03	1.88E-03
T24	G	0.0004	EPC 2, 15, 28	3.55E-03	4.74E-03
T25	F	0.003	EPC 2, 8, 13	5.49E-02	9.54E-02
T26	G	0.0004	EPC 2, 8	7.24E-03	1.01E-02

### Assessed proportions of affect (APOA) of the assigned EPCs

**Fig 5:**
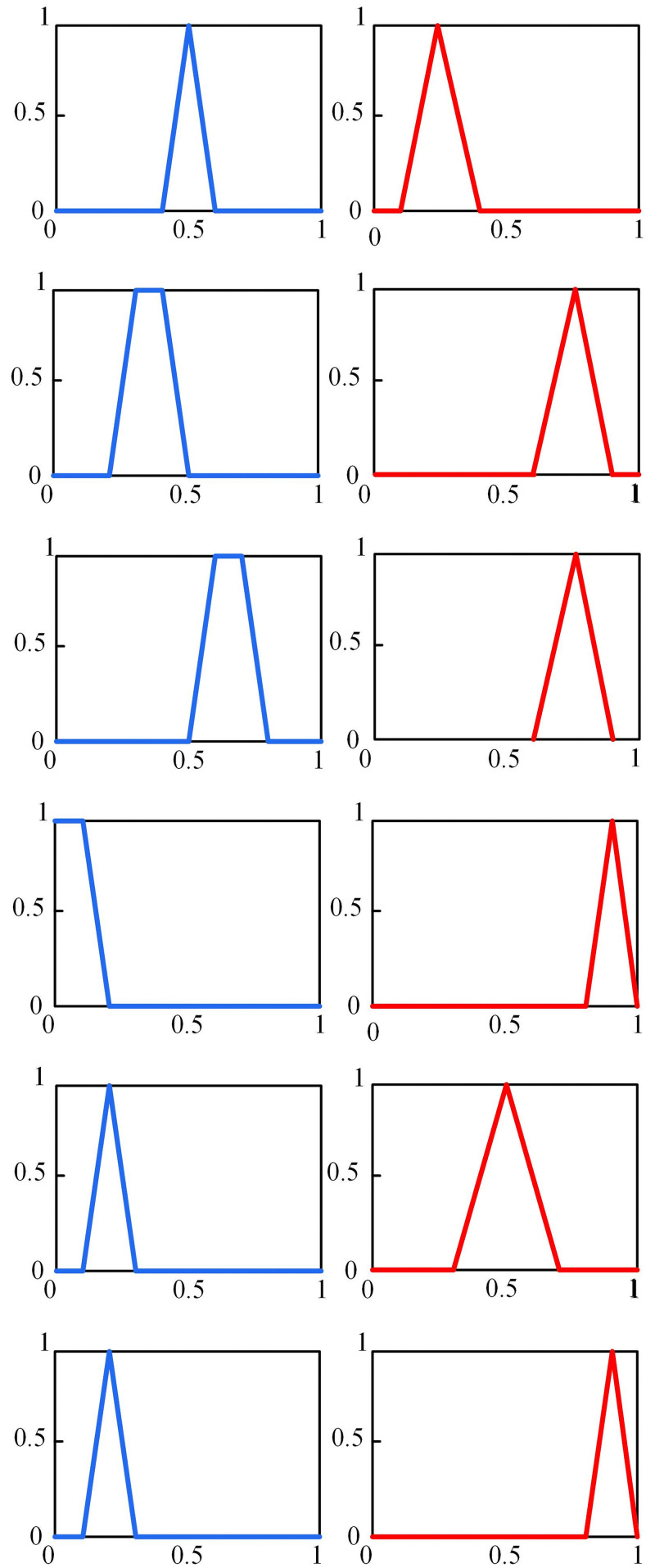
The Z-numbers related to APOA for EPC1 of subtask T1.

According to the employed experts’ opinion, the corresponded Z-numbers of degrading effect of each EPC in relation to subtasks is elicited (for example Z-numbers extracted for EPC1 of subtask T1 are displayed graphically in Fig 5). The right part of Z-numbers is a trapezoid Fuzzy number based on what the experts voted regarding the degrading effect of each EPC. The reliability part of Z-number presents the expert confidence towards their vote. Table 4 presents the Fuzzy number description exploited to describe the linguistic terms for the right and left part in representing Z-numbers for EPC degrading effect evaluation. In electrical distribution practice, as other industries, the expert quality of person increased with the accumulation of education backgrounds and service time. For reasons of anonymity, the identities of the experts who participated in this work have not been wide open, and they have therefore been labeled as E1, E2, E3, E4, E5, and E6. The classical method [47] is employed to obtain a weight to present the expert judgment ability as reported in Table 7. In the next step, the elicited Z-numbers are transformed into normal Fuzzy numbers and aggregate the expert opinion using Eqs 3–5 and 6–11, respectively. Finally, for each EPC a Z-number is formed which is as follows:

$$Z=(\left(a_{1},a_{2},a_{3},a_{4}),(r_{1},r_{2},r_{3})\right)$$

**Table 7:** Expert weighting importance.

No	Job position	Service time	Educational level	Weight factor	Weighting coefficients
E1	Safety engineering (3)	6 to 9 (2)	M.Sc. (4)	9/53	0.17
E2	Electrical technician (2)	10 to 19 (3)	B.Sc. (3)	8/53	0.151
E3	Electrical technician (2)	10 to 19 (3)	Higher national diploma (2)	7/53	0.132
E4	Electrical engineering (3)	6 to 9 (2)	B.Sc. (3)	8/53	0.151
E5	Electrical engineering (3)	10 to 19 (3)	B.Sc. (3)	9/53	0.170
E6	Consultant in electrical system (4)	10 to 19 (3)	PhD (5)	12/53	0.226

In light of the above research method, as an example the EPC1 for the subtask 1 are aggregated and results are tabulated in Table 8, which contains the computations such as similarity degree S(Ai, Aj), average agreement AA(Ei) degree, relative agreement (RAD) degree, etc. Moreover, the similar degree was obtained through all expert opinions, the average agreement, and the relative agreement of other EPC.

**Table 8:** Z-APOA for subtask T1-EPC1 (considered β = 0.5).

Expert	Opinion				Reliability	
E1	M		(0.4, 0.5, 0.5, 0.6)		VL	(0, 0.1, 0.2)
E2	ML		(0.2, 0.3, 0.4, 0.5)		H	(0.6, 0.75, 0.9)
E3	MH		(0.5, 0.6, 0.7, 0.8)		H	(0.6, 0.75, 0.9)
E4	VL		(0, 0, 0.1, 0.2)		VH	(0.8, 0.9, 1.0)
E5	L		(0.1, 0.2, 0.2, 0.3)		M	(0.3, 0.5, 0.7)
E6	L		(0.1, 0.2, 0.2, 0.3)		VH	(0.8, 0.9, 1.0)
• Transform confidence level (α) into the experts’ opinion (using Eq 3)						
E1	0.1		E4	0.9		
E2	0.75		E5	0.5	*E*1 = (0 + 0.1 + 0.2)/3) = 0.1	
E3	0.75		E6	0.9		
• Add weight of confidence level (α) to the experts’ opinions (using Eq 4)						
E1	(0.126, 0.354, 0.158, 0.190)					
E2	(0.173, 0.260, 0.346, 0.433)		$E1=\sqrt{0.1}\times \left(0.4,0.5,0.5,0.6\right)=\left(0.126,0.354,0.158,0.190\right)$			
E3	(0.520, 0.520, 0.606, 0.693)					
E4	(0.00, 0.00, 0.095, 0.190)					
E5	(0.141, 0.141, 0.141, 0.212)					
E6	(0.190, 0.190, 0.190, 0.285)					
S (1–2)	0.872	AA (E1)	0.801	W1	0.170	
S (1–3)	0.497	AA (E2)	0.775	W2	0.151	
S (1–4)	0.819	AA (E3)	0.612	W3	0.132	
S (1–5)	0.936	AA (E4)	0.841	W4	0.151	
S (1–6)	0.882	AA (E5)	0.774	W5	0.170	
S (2–3)	0.625	AA (E6)	0.778	W6	0.226	
S (2–4)	0.691	RA (E1)	0.175	C (E1)	0.172	
S (2–5)	0.808	RA (E2)	0.169	C (E2)	0.160	
S (2–6)	0.881	RA (E3)	0.134	C (E3)	0.133	
S (3–4)	1.000	RA (E4)	0.183	C (E4)	0.167	
S (3–5)	0.433	RA (E5)	0.169	C (E5)	0.169	
S (3–6)	0.506	RA (E6)	0.170	C (E6)	0.198	
S (4–5)	0.883					
S (4–6)	0.810	a1	a2	a3	a4	APOA
S (5–6)	0.810	0.180	0.233	0.241	0.318	0.245

### Compute the final HEP

The corresponding multiplier of the chosen GTT, EPC, and APOA of EPC1 related to subtask 2 inputted into the Eqs (1 and 2) as exhibited in Table 9. Using the same procedure, the HEP values for operation steps of switching of (de-energization) power line are provided in Table 6.

**Table 9:** HEART calculations of EPC1 for subtask 2.

Subtask	GTT	EPC	HERAT effect	Assessed proportion of effect	Assessed effect
T2	0.0004	-A shortage of time available for error detection and correction	11	0.263	(11 − 21) × 0.263) + 1 = 3.63
		-Poor, ambiguous or ill-matched system feedback	4	0.400	(4 − 1) × 0.400) + 1 = 2.2
Degrading factor (‘Total EPC effect assessed’) = 3.63 × 2.2 = 7.986					
Assessed human error probability = 0.0004 × 7.986 = 3.19*E* − 03					

### Sensitivity analysis

**Fig 6:**
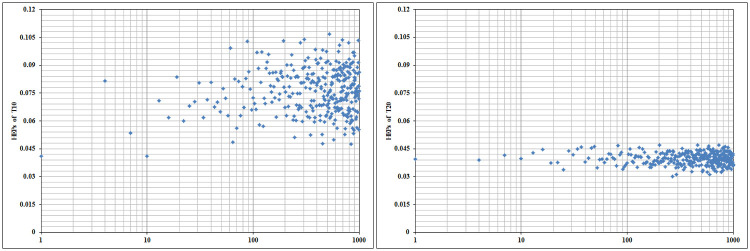
Results of sensitivity analysis of T10 and T20 (logarithmic scale dispersion).

With a view of the attempt will take during the process of expert elicitation, it must be proved using sensitivity analysis on the forehand, if the accuracy of the input parameters is sufficiently affecting the final outcome of the analysis. Therefore, to checking the fruitfulness and robustness of the suggested algorithm of Z-HEART, a sensitivity analysis applied under different possible confidence levels. Each member of expert panel could provide their level of confidence in five linguistic terms as very low (VL), low (L), medium (M), high (H), and very high (VH). So, the set of all possible combinations of level of confidence that may be elicited from six experts would be 15625 cases which about 1000 cases are randomly chosen to display the variation of the HEPs. Towards this purpose, T10 and T20, respectively as the most and least vulnerable subtasks to human error, are taken into account to perform sensitivity analysis. As it reported in Fig 6 the HEP of T10 is widely varied by varying confidence level. Given the random assignment of confidence level in Z-HEART for T10, the maximum HEP is 1.09E-01, and the minimum is equal to 4.11E-02. It is visible that the yielded HEPs are sufficiently apart from each other. Also, in a similar form the maximum and minimum HEP values of the T20 are 4.76E-02 and 3.01E-02, respectively.

Based on the above analysis, it is confirmed that the results are reasonably sensitive to the proper selection of confidence level and hence, the introduced framework in this study can provide suitable contributions in safety measures advice.

## Results and discussion

**Fig 7:**
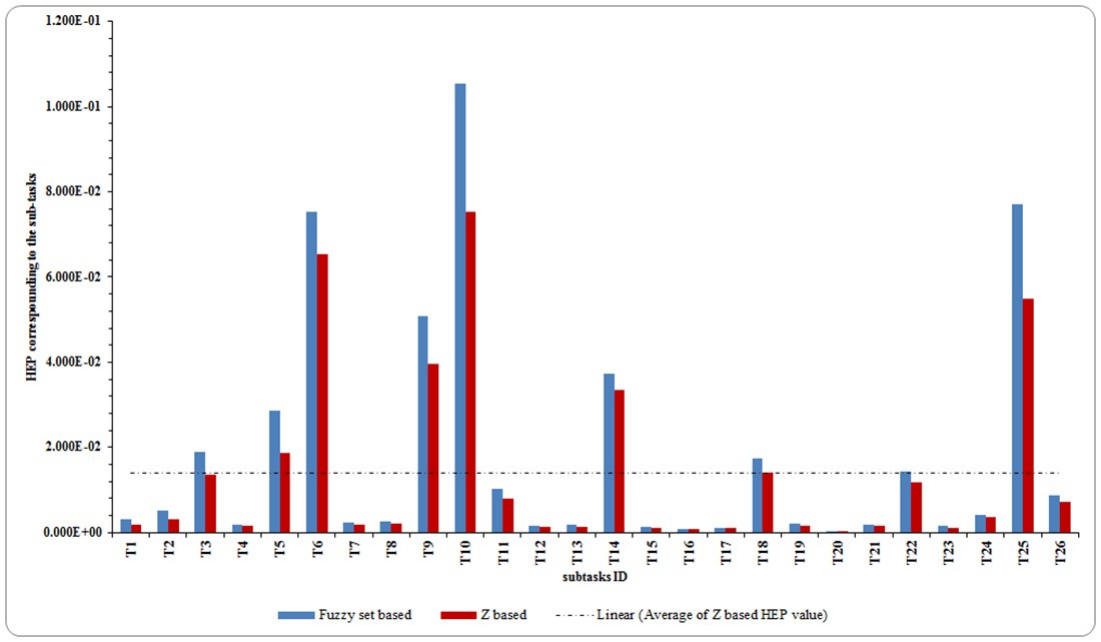
HEP distribution for subtasks of de energization power line.

The results of the implementation of the Z-HEART and Fuzzy HEART for de-energization power line are presented in Table 6.and Fig 7. As can be seen here, there are some differences between HEP derived by Z-HERAT and those calculated by Fuzzy version-based. This is mainly because Fuzzy based algorithms do not take into account the reliability/sureness of propositions. In particular, ignoring reliability of propositions, lead to overestimation of HEP values in HEART Fuzzy based approach. In other words, the Z-HERAT approach considers both objective and subjective reliability of expert evaluation during the APOA estimation process. Existed Fuzzy based approach employed classical Fuzzy values for attributing a linguistic term to a linguistic variable. The reliability of this attribution cannot be set out with the same Fuzzy value (not even type-two Fuzzy sets as they represent the uncertainty in Fuzzy terms themselves instead of the reliability of the propositions) [9].

As the procedure of line de-energization composed of five main tasks with 26 sub-tasks, quantification of human reliability require the total HEP calculation based on sub-task dependency [48]. As can be seen from HTA, DE1 consists of three sequent subtasks (T1- T3) which shall be performed satisfactorily in order to accomplish the authorized de-energization. This implies that, DE1 will fail if any of three subtasks fails. As a result, the HEP value for the DE1 is taken as the maximum value of the three sub-tasks that is 1.36E-02. Analogously, the DE2 will fail in case any of four subtasks (T4- T7) fails. Thus, the HEP value for main task 2 was found to be 6.54E-02. Because of the low dependency among the five subtasks, the HEP value can be assigned 7.53E-02 for DE3 (T8-T12). Moreover, whole main task four, DE4 (T13- T19) fails to operate if any one of its individual subtasks fails (serial system with high dependency). Hence, the HEP value can be yielded as 3.34E-02. Similarly, the same situation was observed in DE5 (T20-T26), with HEP value 5.49E-02. To determine final HEP value for de-energization of power line, five main maintenance tasks should achieve individually (for operation total to succeed). This indicates that de-energization of power line will not be carried out correctly if any of the five main task fails. Numerically, the final value of HEP was obtained to be 7.53E-02.

Reliability by definition is the probability of operating successfully in a given unit of time, and is the automatically the complement to “1” of the probability of cumulative failure. Thus, the equation of *R*(*t*) = 1−*F*(*t*) signifies the relation between cumulative failure *F*(*t*) and reliability *R*(*t*), which are complementary. In other words, the sum of two values is equal to one (or 100%). Therefore, *R*(*t*) = 1 − (6.72*E* − 02) = 9.25*E* − 01, demonstrates that the final HEP value for de-energization of power line is rather high.

In light of the findings, task #T10 (check in no voltage condition) is the most vulnerable subtask in view of human failure in de-energization power line operations, with a HEP of 7.53E-02. The main cause of that is direction of operator attention to other subtasks. Also, this error occurs when the same person disconnect the related feeder and carries out the test voltage. Besides the clearing of distribution line by disconnecting feeder, voltage measurement is a critical task. There are many situations that may induce voltage condition in network, for example malfunction of recloser in the path of a consumer generator, and get back electricity to disconnected network. Moreover, the subtasks #T6 (lock and tag) obtain the second highest HEP value. One of the maintenance practices performed in this company is the lockout/tagout of the energy source. Since the operation may be carried out considerably fast, there could be a time shortage. However, the operator who is in charge to install lockout/tagout at feeder may skip the task in the time allowed. Additionally, the large volume of work resulting may cause inadequate crew relaxation and not enough time with the family, resulting in mental and physical tiredness of the crew. Also, the high HEP value in subtasks #T25 (install temporary ground, also at the other end of de energized section) can be attributed to same reasons.

Accordingly, subtask #20 (sitting the location of inserting ground electrode) has a low contribution to the HEP value. As one can see this subtask is relatively easier in compared with other. It is noteworthy that, among the 26 subtasks, 6 of them had higher HEP value than the average HEP (i.e., 1.41E-02).

## Conclusion

In this paper, we proposed a hybrid human reliability analysis that merged conventional HEART technique with Z-numbers. Although Fuzzy-based HEART technique is an advanced tool for deriving HEP, it does not assist analyst in a concrete manner for calculation of assessed proportion of affect (APOA). Indeed, it ignores the reliability (confidence) of the analyst judgment to a large extent, which may have an effect on the final results. Z-numbers rectify this limitation by taken into account the expert reliability in their judgements, so the Z-numbers employed during APOA estimation in the proposed hybrid approach. It is demonstrated that Z-HEART is feasible for assessing human error and aside from methodological contributions there are many practical benefits for electricity distribution companies in this study. An illustrative example concerning the human error probability was performed for the de-energization of power line. Based on the results, the subtask “check in no voltage condition” is the most vulnerable activity to human error. By observing the obtained results, the overall human reliability value during the whole operation was 9.25E-01. This implied that the reliability of maintenance group was acceptable. Also, the proposed algorithm is a trend toward the formalization of rational decision-making ability of a human expert in uncertainty conditions. Further, validity of the proposed framework was realized through a sensitivity analysis and ensures logical information in development of potential control procedures. Depending on the characteristics of subtasks, control measures can be formulated to eliminate or minimize the risk of unintentional human contributions in electricity distribution companies.

The on-going study has some limitations. The proposed method is heavily relies on domain experts. Hence, produced results from that are only according to the employed expert preferences, which would vary between respondents. Additionally, the findings of the research are based on the opinion elicited from the experts working in the electricity distribution company in Iran.

Our subsequent research goal will focus on the effects of various versions of Fuzzy membership functions for restriction and reliability on the human reliability approximated by the Z-HEART. Moreover, although Z-number has enough power to handle the expert confidence towards their vote, confidence level can be taken into account as an attribute in obtaining specific weightings of selected experts using Fuzzy analytic hierarchy process (FAHP) as well. We plan to conduct the FAHP version-based HEART, followed by making a lucid comparison between the computed results from the present study and HEART-FAHP based results.
